# Supplementary material for: Content-rich biological network constructed by mining PubMed abstracts
Source: BMC Bioinformatics. 2004 Oct 8;5:147. doi: 10.1186/1471-2105-5-147 (PMC528731; doi:10.1186/1471-2105-5-147)
Supplement: Additional File 2 — The original results of the above study (non-essential files are deleted to keep the file size under the limit set by BMC bioinformatics). [file 1471-2105-5-147-S2.bz2 › chilibotAdditionalFile2/dip20/468ID9660793E10524/html/CCL11_CXCR3.html]

 


 **CCL11** and **CXCR3** 
  
Found 87 abstracts in PubMed, retrieved 20.  
 

 What does Google say? 
 PDF only 
| .edu only 

---

**Interactive relationship** (e.g. stimulation, inhibition, etc)

**Neutral relationship**- Here, we report that although  **CCL11** , CCL24 and CCL26 are neither agonists nor antagonists of  **CXCR3** ,  **CCL11**  binds with high affinity to  **CXCR3** .  Ref: 12884299 Eur J Immunol, 2003
- N terminal domain of eotaxin  [ **CCL11** ]  3 is important for activation of CC chemokine receptor 3  [ **CXCR3** ] .  Ref: 12538912 Protein Eng, 2002
- This suggests that, in vivo,  **CXCR3**  may act as a decoy receptor, sequestering locally produced  **CCL11** .  Ref: 12884299 Eur J Immunol, 2003
- Eotaxin  [ **CCL11** ]  is a potent chemokine that acts via CC chemokine receptor 3  [ **CXCR3** ]  CCR3 to induce chemotaxis, mainly on eosinophils.  Ref: 12413953 Biochem Biophys Res Commun, 2002
- Eotaxin  [ **CCL11** ] , an inducer of eosinophil migration and activation, exerts its activity by binding to CCR3, the C C chemokine receptor 3  [ **CXCR3** ] .  Ref: 12857386 J Biomol Screen, 2003

**Non-interactive relationship** (e.g. studied together, co-existance, homology, etc.)

- An eosinophil active chemokine, eotaxin  [ **CCL11** ] , and its receptor, C C chemokine receptor 3  [ **CXCR3** ] , are particularly attractive as novel targets of immunological intervention for the disease.  Ref: 12842759 Cytokine< MedlineTA>Cytokine, 2003
- To study mechanisms of progenitor cell mobilization in allergic reactions, we investigated fluctuations in the expression of the eotaxin  [ **CCL11** ]  receptor, CC chemokine receptor 3  [ **CXCR3** ]  CCR3, on CD34 cells from stable asthmatics following allergeni.e. antigen challenge.  Ref: 12871220 ImmunologyImmunology, 2003
- Type 1 chemokines CXC chemokine ligand CXCL 9 and CXCL10 monokine induced by IFN gamma and IFN gamma inducible protein of 10 kDa  **CXCR3**  ligands, and type 2 chemokines CC chemokine ligand CCL 11  [ **CCL11** ]  eotaxin  [ **CCL11** ]  CCR3 ligand, CCL17 thymus and activation regulated chemokine CCR4 ligand and CCL22 monocyte derived chemokine CCR4 ligand were readily detectable in the plasma of most participants.  Ref: 12407016 Int Immunol, 2002
- Xenograft rejection was associated with early intragraft transcript expression for monocyte chemotactic protein 1 MCP 1 3 to 5 days, IP 10  [ **CXCR3** ]  3 to 4 days and macrophage inflammatory protein 1alpha MIP 1alpha 3 to 5 days and subsequent expression of eotaxin  [ **CCL11** ]  days 4 to 10, MIP 1beta days 4 and 5 and regulated on activation, normal T cell expressed and secreted RANTES days 4 to 6 mRNA.  Ref: 12588649 Xenotransplantation, 2003
- In particular, the eotaxin  [ **CCL11** ]  subfamily of chemokines and their receptor CC chemokine receptor 3  [ **CXCR3** ]  have emerged as central regulators of the asthmatic response.  Ref: 12589338 J Allergy Clin Immunol, 2003
- The CXC chemokines such as IL 8, IP10  [ **CXCR3** ] , Mig, SDF 1 alpha or CC chemokines such as MCP 1, MIP 1 alpha, eotaxin  [ **CCL11** ] , RANTES have been frequently harvested from tumor tissues or the biological fluids of patients.  Ref: 12489485 Recenti Prog Med, 2002
- This pattern was consistent with the early recruitment of macrophages MCP 1, MIP 1alpha, the influx of CD4 T cells MCP 1, MIP 1alpha, MIP 1beta, IP 10  [ **CXCR3** ]  and RANTES and the characteristic infiltrate of eosinophils eotaxin  [ **CCL11** ]  and RANTES associated with islet xenograft rejection.  Ref: 12588649 Xenotransplantation, 2003
- Despite there being no difference in expression of CC chemokine receptor 3  [ **CXCR3** ] , the priming effect of RANTES and eotaxin  [ **CCL11** ]  was significantly enhanced in eosinophils from the patients.  Ref: 12797483 Eur Respir J, 2003
- Biopsies were stained for CD68, CD3, elastase, eotaxin  [ **CCL11** ] , IP 10  [ **CXCR3** ] , MCP 1, MCP 3, and IL 8 and analyzed by NIH Image analyzer.  Ref: 12827410 Int J Colorectal Dis< MedlineTA>Int J Colorectal Dis, 2003
- Consistent with these results, infected cells from WT mice preferentially migrated in response to IP 10  [ **CXCR3** ]   **CXCR3**  ligand, while those from GKO mice migrated in response to eotaxin  [ **CCL11** ]   **CCL11**  CCR3 ligand.  Ref: 12875978 Am J Pathol, 2003
- Hence, eosinophil responses mediated by chemokines acting at CCR3 may be regulated by two distinct mechanisms the antagonistic effects of  **CXCR3**  ligands and the sequestration of  **CCL11**  by  **CXCR3**  expressing cells.  Ref: 12884299 Eur J Immunol, 2003
